# From Organizational Justice Perceptions to Turnover Intentions: The Mediating Effects of Burnout and Job Satisfaction

**DOI:** 10.5964/ejop.v14i3.1490

**Published:** 2018-08-31

**Authors:** Juan Diego Vaamonde, Alicia Omar, Solana Salessi

**Affiliations:** a National Scientific and Technical Research Council (CONICET) - Research Institute, School of Humanities and Arts, National University of Rosario, Rosario, Argentina; b School of Psychology, National University of Rosario, Rosario, Argentina; c Department of Society, State, and Government, National University of Rafaela, Rafaela, Argentina; d Department of Culture, Education, and Knowledge, National University of Rafaela, Rafaela, Argentina; Department of Psychology, Webster University Geneva, Geneva, Switzerland; Glasgow Caledonian University, Glasgow, United Kingdom

**Keywords:** organizational justice, burnout, job satisfaction, turnover intentions, Argentine employees

## Abstract

Turnover intentions (TI) stand as an insidious problem that impacts on the functioning of organizations and the well-being of their members. Currently, there is a growing interest in identifying the explanatory mechanisms of TI, in order to strengthen and retain valued employees for organizations. In line with this trend, the aim of the present study was to test an integrative serial multiple mediation model that examined the possible mediating role of burnout and job satisfaction in the relationships between organizational justice and TI. To achieve this objective, a cross-sectional empirical study was carried out on a multi-occupational sample of 408 Argentine employees (219 women and 189 men). Participants completed a self-report questionnaire comprising previously validated measures for the target population. Structural equation modeling showed that perceptions of distributive, procedural, and interpersonal justice have negative indirect effects on TI through burnout and job satisfaction, while perceptions of informational justice exert such effects on TI only through job satisfaction. These results indicate that distributive, procedural, and interpersonal justice perceptions relate to lower levels of burnout, which in turn promote greater job satisfaction and lower TI among employees. In addition, informational justice perceptions are positively related to job satisfaction, leading to a decrease in employees’ TI. Findings are discussed in light of their theoretical and practical implications. Managers and human resource professionals could consider the research results in their attempts to design and implement talent retention strategies within organizations.

The last decades have witnessed profound changes in the world of work. The globalization of economies, together with market liberalization and the emergence of new recruitment and employment rules, has created favorable conditions for labor instability and employee turnover (International Labour Organization, 2016). At the organizational level, turnover of qualified employees leads to poor performance and high costs of recruitment, selection, and training of new staff; while, at the individual level, turnover affects the levels of commitment, identification, and social integration of workers (Chang, Wang, & Huang, 2013). In order to prevent and intervene in this issue, researchers and organizational specialists have turned to the study of *turnover intentions* (TI), defined as employees’ conscious and deliberate willingness to leave their organizations (Allisey, Noblet, Lamontagne, & Houdmont, 2014; Chang et al., 2013; Flint, Haley, & McNally, 2013). Over the last years, employees’ TI have occupied a prominent position in human resource management and organizational research, mainly because they stand as a strong predictor of actual employee turnover (Chang et al., 2013; Tett & Meyer, 1993).

Recent empirical evidence indicates that TI are an insidious phenomenon that has negative consequences for both workers and organizations, such as absenteeism, frustration, low motivation, and reduced work performance (Flint et al., 2013; Han, Han, An, & Lim, 2015). In view of this situation, and given the strategic value attributed to the retention of human capital, there is now a growing interest in identifying the explanatory variables of workers’ TI. In this regard, although organizational commitment, occupational stress, and job satisfaction are among the most studied predictors of these withdrawal cognitions (Chang et al., 2013), little is known about the role that variables such as organizational justice perceptions or burnout syndrome could play in TI of Latin American workers in general, and Argentine workers in particular. Moreover, while early studies of TI generally focused on their external, work-related and personal correlates, recent research has shifted the focus onto the examination of different models of TI, trying to identify factors that might mediate or moderate the relationships between certain predictor variables and TI (Boroş & Curşeu, 2013; Flint et al., 2013; Suurd Ralph & Holmvall, 2016). In line with this current trend, this paper aims to examine an integrative serial multiple mediation model that posits the possible mediating role of burnout and job satisfaction in the relationships between organizational justice and TI. The study contributes to the literature in two significant ways. First, it extends past research by exploring the potential indirect effects of organizational justice on TI. Second, by examining burnout and job satisfaction as serial mediators in the organizational justice–TI relationship, the study contributes to the understanding of the underlying psychological mechanisms through which organizational justice perceptions influence TI of employees.

## Literature Review and Hypotheses

### Turnover Intentions (TI)

Employee turnover represents one of the most expensive problems of today’s organizations to the point that the cost of a resigning employee equates about a year of her/his salary (Boroş & Curşeu, 2013). Undoubtedly, high rates of voluntary turnover are often harmful to organization performance and work outcomes (Flint et al., 2013). In this sense, given that TI constitute the final step of a series of withdrawal cognitions and/or behaviors, they are considered a useful variable to anticipate and prevent employee turnover (Chang et al., 2013; Flint et al., 2013; Tett & Meyer, 1993). Besides, even when TI do not lead to actual turnover, they have a negative impact on organizational effectiveness, as employees with unfulfilled TI are likely to engage in other kinds of withdrawal behaviors (Ferreira, Martinez, Lamelas, & Rodrigues, 2017). Thus, as Chang et al. (2013, p. 2) stated, “if antecedents of TI can be identified in advance, firms can develop appropriate interventions to enhance competitive advantage and prevent avoidable visible and invisible costs”.

The literature review indicates that one potential useful antecedent of TI are organizational justice perceptions. However, empirical research on the relationship between these variables is still scarce. In fact, a search in the databases Academic Search Premier, Psychology and Behavioral Sciences Collection, and PsycINFO, with *turnover* and *justice* as general subject terms, yields a total of only 134 papers published between 1991 and 2017, and none of these studies has been carried out in Latin America or the Caribbean. This is rather surprising given the abundance of evidence (e.g., International Labour Organization, 2015) claiming the importance of identifying determinants of turnover so as to boost employee retention and organizational performance. Consequently, the general purpose of this research was to contribute to fill this gap by analyzing the relationships between organizational justice and TI among Argentine employees.

### Organizational Justice and TI

*Organizational justice* refers to employees’ perceptions of what is fair and what is unfair within their organizations (Colquitt, 2001). It has been defined as a multidimensional construct composed of four distinct factors (Colquitt et al., 2013): *distributive justice* (perceived fairness related to outcomes and distributions), *procedural justice* (perceived fairness of procedures used to determine outcome distributions), *interpersonal justice* (quality of interpersonal treatment received when procedures are implemented), and *informational justice* (level of adequacy, honesty, and convenience of information conveyed about why procedures are used a certain way or how outcomes are determined). Organizational justice has captured the attention of experts due to its effects on a wide repertoire of employee attitudes, cognitions, and behaviors towards the organizations and their members (Colquitt et al., 2013; Silva & Caetano, 2016).

Previous studies (Campbell, Perry, Maertz, Allen, & Griffeth, 2013; Kim & Kao, 2014; Silva & Caetano, 2016; Suurd Ralph & Holmvall, 2016) have shown that justice perceptions promote higher organizational commitment, higher job satisfaction, and lower TI. This observation finds support in social exchange theory (Blau, 1964), which posits that if employees perceive benefits in their work exchanges, they are likely to continue to participate in them; if not, if there is little compensation or unfair organizational treatment, employees are likely to avoid future exchanges, and one form of avoidance involves resorting to withdrawal cognitions and/or behaviors (Flint et al., 2013). So, based on these antecedents, we hypothesize that:

*Hypothesis 1:* organizational justice perceptions (i.e. distributive, procedural, interpersonal, and informational justice) will be negatively related to TI (*H1*).

To date, the limited research available on the linkages between organizational justice and TI has focused almost entirely on their direct relationships, neglecting the study of possible mediating variables. In an attempt to address this limitation, Flint et al. (2013) reported that justice perceptions exert an indirect effect on TI of Canadian employees through their organizational and supervisory commitment. Tayfur, Karapinar, and Camgoz (2013) found that emotional exhaustion and cynicism mediated the relationships between perceptions of justice and TI of Turkish employees. More recently, Suurd Ralph and Holmvall (2016) observed that overall justice and strain mediated the relationships between distributive, procedural, and interpersonal justice and TI of Canadian Armed Forces personnel. Despite this empirical evidence, no research has so far examined the possible serial mediating effects of burnout and job satisfaction in the relationships between organizational justice and TI.

### Burnout and Job Satisfaction as Mediators Between Organizational Justice and TI

The term *burnout* describes a syndrome characterized by emotional exhaustion, cynicism or depersonalization, and reduced or lack of personal accomplishment, which results from long-term exposure to emotionally demanding stressors in the workplace (Leka & Jain, 2010; Maslach & Jackson, 1986). Such stressors exceed individuals’ coping abilities and can ultimately affect their health in a negative way. Although the study of burnout was initially confined to caring and social professions (e.g., nurses, doctors, dentists, social workers, and teachers), researchers have expanded its analysis to include a much broader spectrum of workers (Leka & Jain, 2010).

Previous research (Campbell et al., 2013; Moreno-Jiménez, Gálvez-Herrer, Rodríguez-Carvajal, & Sanz Vergel, 2012; Tayfur et al., 2013) has shown that burnout develops as employees find themselves immersed in situations of chronic stress, including work environments where low organizational justice prevails. Employees who perceive constant unfair treatment at work are likely to feel emotionally exhausted, to undervalue their abilities and achievements, and to develop cynical attitudes toward their organization and coworkers. This increase in burnout levels can lead, in turn, to higher TI among employees (Han et al., 2015; Moreno-Jiménez et al., 2012). So, considering these antecedents and arguments, we propose the following hypothesis:

*Hypothesis 2:* burnout will be negatively related to organizational justice perceptions (*H2a*) and positively related to TI (*H2b*).

A wide diversity of studies show that burnout has a clear negative impact on *job satisfaction* (Leka & Jain, 2010; Scanlan & Still, 2013), which is usually defined as an attitude toward work experiences that includes cognitive and emotional aspects and has important implications for both employee behavior and organizational outcomes (Moura, Orgambídez-Ramos, & Gonçalves, 2014; Salessi & Omar, 2016). Individuals suffering from emotional exhaustion, depersonalization, and/or lack of personal accomplishment at work will, understandably, experience job dissatisfaction, increasing their chances of developing TI (Kuo, Lin, & Li, 2014; Salessi & Omar, 2016). These withdrawal cognitions emerge as a coping mechanism triggered by the unpleasant situation, in a failed endeavor to elude it. In fact, over the years, numerous studies have examined the relationship between job satisfaction and labor turnover (e.g., Allisey et al., 2014; Kim & Kao, 2014; Kuo et al., 2014; Scanlan & Still, 2013; Zeffane & Bani Melhem, 2017), concluding that satisfied employees present low or no TI. Therefore, based on these antecedents, we posit the following hypothesis:

*Hypothesis 3:* job satisfaction will be positively related to organizational justice perceptions (*H3a*) and negatively related to burnout (*H3b*) and TI (*H3c*).

As for the mediating roles of burnout and job satisfaction, only a handful of studies have recently started to examine these roles in TI explanatory models. In this sense, regarding burnout, Gabel Shemueli, Dolan, Suárez Ceretti, and Nuñez del Prado (2016) found that it fully mediated the relationship between work overload and TI of nurses from Spain and Uruguay. In a similar vein, Amponsah-Tawiah, Annor, and Arthur (2016) observed, in a multi-occupational Ghanaian sample, that commuting stress was indirectly related to job satisfaction and TI via burnout. Also, based on a sample of Pakistani engineers and supervisors, Shaukat, Yousaf, and Sanders (2017) used structural equation modeling to prove that burnout mediated the association between relationship conflict and different outcome variables, including TI. Regarding job satisfaction as a mediator variable, Kuo et al. (2014) showed that it significantly mediated the relationship between work stress and TI among long-term care nurses in Taiwan. Also, drawing on data from a sample of Chinese nurses, Feng, Su, Yang, Xia, and Su (2017) observed that social status exerted significant indirect effects on TI through job satisfaction. Finally, Ferreira et al. (2017) conducted a multilevel study with Portuguese hotel employees in which they confirmed that job satisfaction and job embeddedness fully mediated the relationship between different task characteristics and TI.

The mediating roles of burnout and job satisfaction in the relationship between organizational justice and TI could be explained from the perspective of the conservation of resources theory (Hobfoll, 1989), which states that individuals seek to acquire and maintain resources (objects, characteristics, conditions, and/or energies) and when there is a loss (or a threat of a loss) of such resources, they become stressed. In the context of this study, perceived low organizational justice is considered a stressor that threats individual resources (e.g., salary, time, fair working conditions, etc.). This situation could promote over time higher levels of burnout, leading to lower job satisfaction and greater TI among employees.

**Figure 1:**
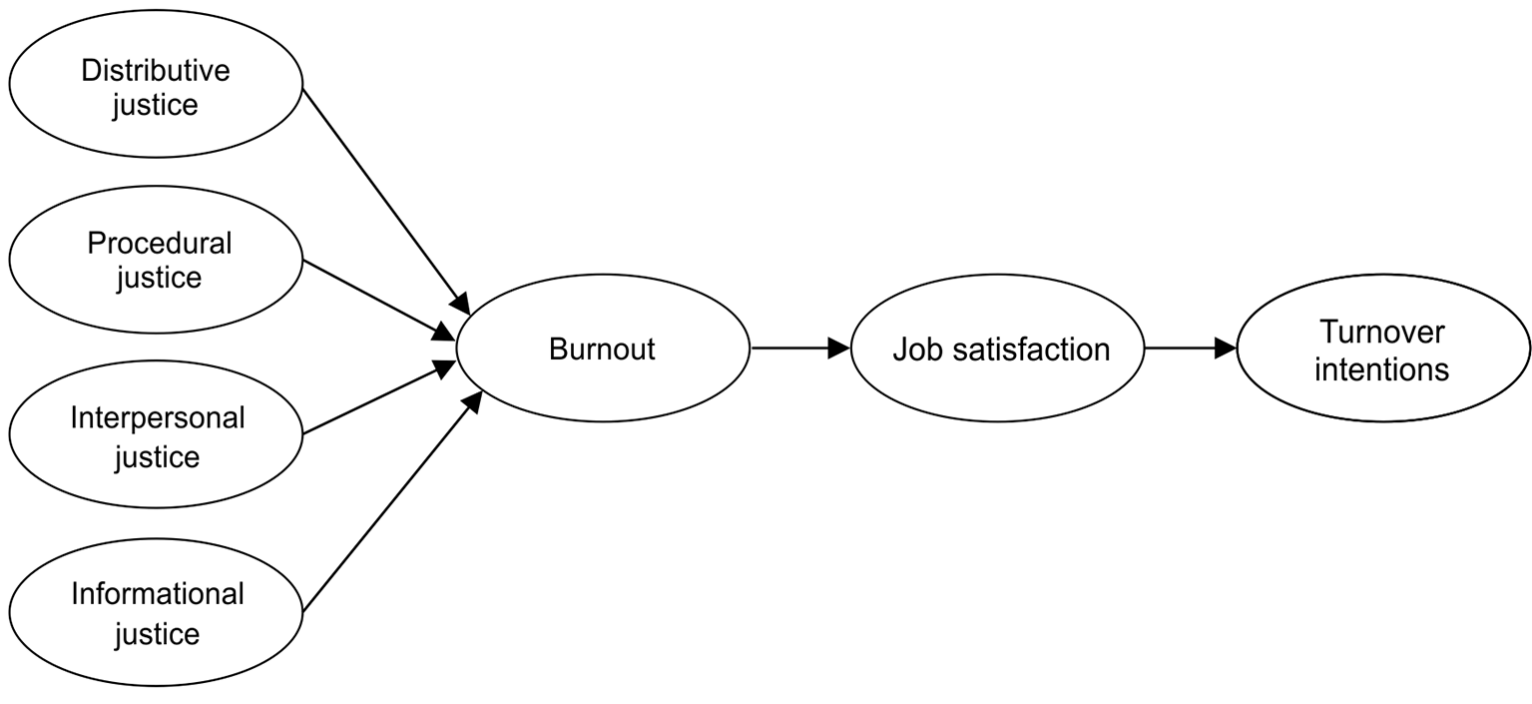
Proposed serial mediation model.

So, in spite of the fact that conclusive evidence indicates, on the one hand, that low justice perceptions produce higher burnout and lower job satisfaction, and, on the other hand, that a decrease in job satisfaction generates a breeding ground for the development of employees’ TI (Allisey et al., 2014; Chang et al., 2013; Feng et al., 2017; International Labour Organization, 2016; Scanlan & Still, 2013; Shaukat et al., 2017; Tayfur et al., 2013), no research has yet explored the possible links between organizational justice, burnout, job satisfaction, and TI in a single integrative model. Therefore, in light of these theoretical and empirical antecedents, and considering the insufficiency of the current state of knowledge to account for TI, the aim of the present study was to analyze the underlying mechanisms of the relationship between organizational justice perceptions and TI of Argentine employees. Specifically, we tested a serial multiple mediation model in which justice perceptions and TI were set as predictor and criterion variables, respectively, while burnout and job satisfaction played the role of mediating variables (Figure 1). The main hypothesis of this model was as follows:

*Hypothesis 4:* burnout and job satisfaction will act as serial mediators in the relationships between organizational justice perceptions (i.e. distributive, procedural, interpersonal, and informational justice) and TI (*H4*).

## Method

### Design

The study adopted an empirical, cross-sectional research design. It was framed within the guidelines of the associative-explanatory strategy (Ato, López, & Benavente, 2013), since its purpose was to explore the functional relationships between a particular group of variables to predict or explain their behavior. According to Ato and his colleagues (2013), three characteristics allow to operationally define this type of non-experimental study: (a) the existence of a single sample of participants that is not usually randomly selected; (b) the measurement of two or more variables that are generally of a quantitative nature; and (c) the availability of a correlation or a covariance matrix for the statistical analysis. In this particular research, the explanatory design is composed of a measurement and a structural model, with both latent and observed variables. These models are known as *structural equation models (SEM)*.

### Participants

Four hundred and eight employees (219 women and 189 men) of public and private organizations located in the central region of Argentina participated in the study. Their mean age was 29.4 years (*SD* = 9.2) and the mean tenure was 4.7 years (*SD* = 5.5). Regarding their educational level, 34.8% had secondary education, 39.5% had tertiary education, and the remaining 25.7% had university education. In relation to the organizational sector, 36.3% worked in public organizations, and 63.7% worked in private companies. As far as organizational activity is concerned, 38.5% belonged to trade/business companies, 21.6% to educational institutions, 19.3% to industries, 15.7% to public administration, and the remaining 4.9% to health organizations. While 41.7% and 54.2% were hired and permanent employees, respectively, 4.1% were managers or supervisors.

### Measures

#### Organizational Justice

Organizational justice was explored using the Argentine adaptation (Vaamonde & Salessi, 2014) of Colquitt’s Organizational Justice Scale (2001). This measure consists of 20 items that explore the dimensions of distributive justice (4 items, α = .90, e.g., “my outcomes [salary, promotions, rewards, etc.] reflect the effort I have put into my work”), procedural justice (7 items, α = .87, e.g., “the procedures that my organization uses to make decisions and arrive at my outcomes have been applied consistently”), interpersonal justice (4 items, α = .92, e.g., “my boss/supervisor/manager has treated me in a polite manner”), and informational justice (5 items, α = .90, e.g., “my boss/supervisor/manager has provided me with reasonable explanations regarding the procedures enacted”). The items were assessed using a 5-point Likert scale ranging from 1 (*never*) to 5 (*always*). The instrument has proven to have good validity indices (χ^2^/*df* = 2.36, PGFI = .61, GFI = .91, CFI = .94, RMSEA = .03). The original average variance extracted (AVE) values (all above .60), as well as their square roots, demonstrate adequate convergent and discriminant validity.

#### Burnout

The Latin American validation (González-Llaneza, 2007) of the Maslach Burnout Inventory (Maslach & Jackson, 1986) was utilized to assess burnout syndrome. This instrument is composed of 22 items distributed in three dimensions: emotional exhaustion (9 items, α = .87, e.g., “I feel emotionally drained from my work”), depersonalization (5 items, α = .73, e.g., “I feel I treat some people at my work as if they were impersonal objects”), and reduced or lack of personal accomplishment (8 reverse scored items, α = .72, e.g., “I have accomplished many worthwhile things in this job”). A 5-point Likert scale ranging from 1 (*strongly disagree*) to 5 (*strongly agree*) was used to assess these dimensions. The inventory presents adequate validity indices (χ^2^ = 224.63, *p* < .001, GFI = .92, AGFI = .89, CFI = .93, RMSEA = .07) and ample evidence of convergent and discriminant validity across different Spanish-speaking contexts (Gil-Monte, 2005; Martínez Pérez, 2010; Olivares-Faúndez, Mena-Miranda, Macía-Sepúlveda, & Jélvez-Wilke, 2014).

#### Job Satisfaction

The Argentine validation (Salessi & Omar, 2016) of the Job Satisfaction Scale (Macdonald & MacIntyre, 1997) was used to examine this variable. This measure explores job satisfaction as a single factor through 7 items (α = .82, e.g., “I feel good working for this organization”) with a 5-point Likert scale (1 = *strongly disagree* to 5 = *strongly agree*). Confirmatory factor analysis has shown remarkable validity indices for this instrument (χ^2^ = 18.79, *p* = .07, GFI = .99, AGFI = .97, CFI = .99, RMSEA = .04). Convergent/discriminant validity was originally assessed through correlations between the scores on job satisfaction and other theoretically related constructs such as commitment and trust, achieving good psychometric results (Salessi & Omar, 2016).

#### Turnover Intentions

TI were measured using the Turnover Intentions Scale developed by Vaamonde (2015). This instrument consists of 7 items that explore the construct as a single factor (α = .91, e.g., “I am thinking of leaving my job”) with a 5-point Likert scale (1 = *strongly disagree* to 5 = *strongly agree*). The measure exhibits satisfactory validity indices (χ^2^ = 1.11, *p* = .35, GFI = .98, AGFI = .96, CFI = .99, RMSEA = .02) as well as convergent and discriminant validity (AVE > .60; √AVE greater than inter-construct correlations).

#### Demographic Data

The questionnaire included a final section to collect the following demographic data: gender, age, educational level, tenure, position, organizational sector, and organizational activity.

### Procedures

All participants were contacted at their workplaces and informed of the objectives of the study. After this introduction, they were invited to answer a booklet containing a sheet of informed consent, a set of detailed instructions, and the instruments described above. Data collection took place during working hours at the physical spaces provided by the organizations for this purpose. Participation was anonymous and voluntary, and no incentives were offered for completing the survey.

With regard to ethical issues, the study was reviewed and approved by the Research Ethics Committee of the National University of Rosario, Argentina (Resolution C.S. nº 206/2015). It was therefore conducted in full compliance with the research ethical standards recommended not only by the National University of Rosario and the CONICET, Argentina, but also by the American Psychological Association.

### Data Analysis

First, preliminary analyses were performed to examine the presence of missing values, outliers, and influential observations, such as skewness, kurtosis, and multicollinearity (Hair, Black, Babin, & Anderson, 2010). Second, a confirmatory factor analysis was carried out to assess the measurement model. Third, descriptive statistics and bivariate correlations were calculated between the variables of interest. Fourth, the proposed theoretical model was tested by means of structural equation modeling. The strategy of rival models was used to contrast two mediation models. To evaluate the goodness of fit, the following tests and indices were calculated: chi-square (χ^2^), χ^2^/degrees of freedom ratio (χ^2^/*df*), comparative fit index (CFI), Tucker-Lewis index (TLI), parsimony normed fit index (PNFI), root mean standard error of approximation (RMSEA), and the closeness of fit for RMSEA (PCLOSE, *p* of Close Fit). Adequate goodness-of-fit is indicated by χ^2^/*df* less than 3, CFI and TLI equal to or greater than .90, PNFI greater than .50, RMSEA less than .05, and PCLOSE greater than .50 (Byrne, 2016; Hair et al., 2010). Besides, when comparing alternative models with different degrees of freedom, the one with the highest PNFI presents the highest level of parsimonious fit. Finally, the indirect effects of organizational justice on TI through burnout and job satisfaction were examined using bootstrap analysis, selecting 5000 random samples and 95% bias-corrected confidence intervals. These analyses were performed using SPSS 20 and AMOS 20.

## Results

### Preliminary Data Analyses

Given that less than 2% of missing values were detected, median imputation was used for their replacement. Skewness and kurtosis statistics did not exceed the critical limits of ±2.0 (Byrne, 2016; Hair et al., 2010). Mahalanobis and Cook’s distances did not reveal the presence of outliers or influential observations, and no multicollinearity problems were found among the study variables. Taken together, these results supported the implementation of structural equation modeling with maximum likelihood estimation method.

### Measurement Model Analysis

A confirmatory factor analysis was conducted to assess the measurement model. Organizational justice, job satisfaction, and TI were estimated as correlated first-order factors, with their respective items as observable variables. In order to achieve greater model parsimony (Byrne, 2016), burnout was operationalized as a second-order latent construct, with its corresponding dimensions, indicators, and interfactor correlations. To optimize the initial model fit (Table 1), and considering the theoretical basis for the different constructs, those items with standardized factor loadings lower than .60 were deleted, leading to final loadings ranging from .60 to .92 (*p* < .01). As can be seen in Table 1, the fit indices obtained for this revised model were very satisfactory. Furthermore, with the purpose of assessing construct reliability and validity, Cronbach’s alpha coefficients and composite reliability (CR), average variance extracted (AVE), and square root of AVE were calculated for each factor. Cronbach’s alphas and CR equal to or greater than .70, AVE ​​equal to or greater than .50, and square roots of AVE greater than inter-construct correlations are considered sufficient evidence of reliability, convergent validity, and discriminant validity, respectively (Hair et al., 2010; Henseler, Ringle, & Sarstedt, 2015). As shown in Table 2, all these reliability and validity analyses yielded expected results. Harman’s one-factor test was also performed to rule out common method biases (Podsakoff, MacKenzie, & Podsakoff, 2012).

**Table 1:** Goodness-of-Fit Statistics for the Estimated Measurement Models

Model	χ^2^	*df*	χ^2^/*df*	CFI	TLI	PNFI	RMSEA	PCLOSE
Initial measurement model	2696.81**	1454	1.86	.90	.90	.77	.05	.99
Revised measurement model	1197.43**	675	1.77	.95	.95	.81	.04	.99

*Note.* CFI = comparative fit index; TLI = Tucker-Lewis index; PNFI = parsimony normed fit index; RMSEA = root mean standard error of approximation (RMSEA); PCLOSE = *p* of close fit.

**p < .001.

**Table 2:** Reliability and Validity Indices of the Measures

Measure	*Cronbach’s* α	CR	AVE	√ AVE
1. Distributive justice	.93	.93	.76	.87
2. Procedural justice	.86	.87	.52	.72
3. Interpersonal justice	.87	.89	.67	.82
4. Informational justice	.92	.91	.68	.82
5. Burnout	.85	.76	.54	.73
6. Job satisfaction	.83	.81	.52	.72
7. TI	.92	.92	.62	.79

*Note.* TI = turnover intentions; CR = composite reliability; AVE = average variance extracted.

### Descriptive Statistics and Bivariate Correlations

Table 3 presents the means, standard deviations, and product-moment correlations coefficients for the variables of interest. As expected, all facets of organizational justice perceptions (i.e. distributive, procedural, interpersonal, and informational justice) were negatively related to both TI (*H1*) and burnout (*H2a*), and positively related to job satisfaction (*H3a*), while TI were positively associated with burnout (*H2b*) and negatively associated with job satisfaction (*H3c*). Burnout also appeared to be negatively linked to job satisfaction (*H3b*). Therefore, Hypotheses 1 to 3 were fully supported.

**Table 3:** Descriptive Statistics and Bivariate Correlations Between the Variables Under Study (n = 408)

Variable	*M*	*SD*	1	2	3	4	5	6	7
1. Distributive justice	2.68	1.19	-						
2. Procedural justice	3.17	1.01	.50**	-					
3. Interpersonal justice	4.18	0.95	.33**	.48**	-				
4. Informational justice	3.69	1.16	.44**	.64**	.66**	-			
5. Burnout	2.47	0.65	-.47**	-.51**	-.47**	-.48**	-		
6. Job satisfaction	3.43	0.78	.54**	.57**	.44**	.56**	-.56**	-	
7. TI	2.79	1.14	-.40**	-.44**	-.30**	-.39**	.49**	-.66**	-

*Note.* TI = turnover intentions.

**p < .001.

### Structural Model Analysis

**Figure 2:**
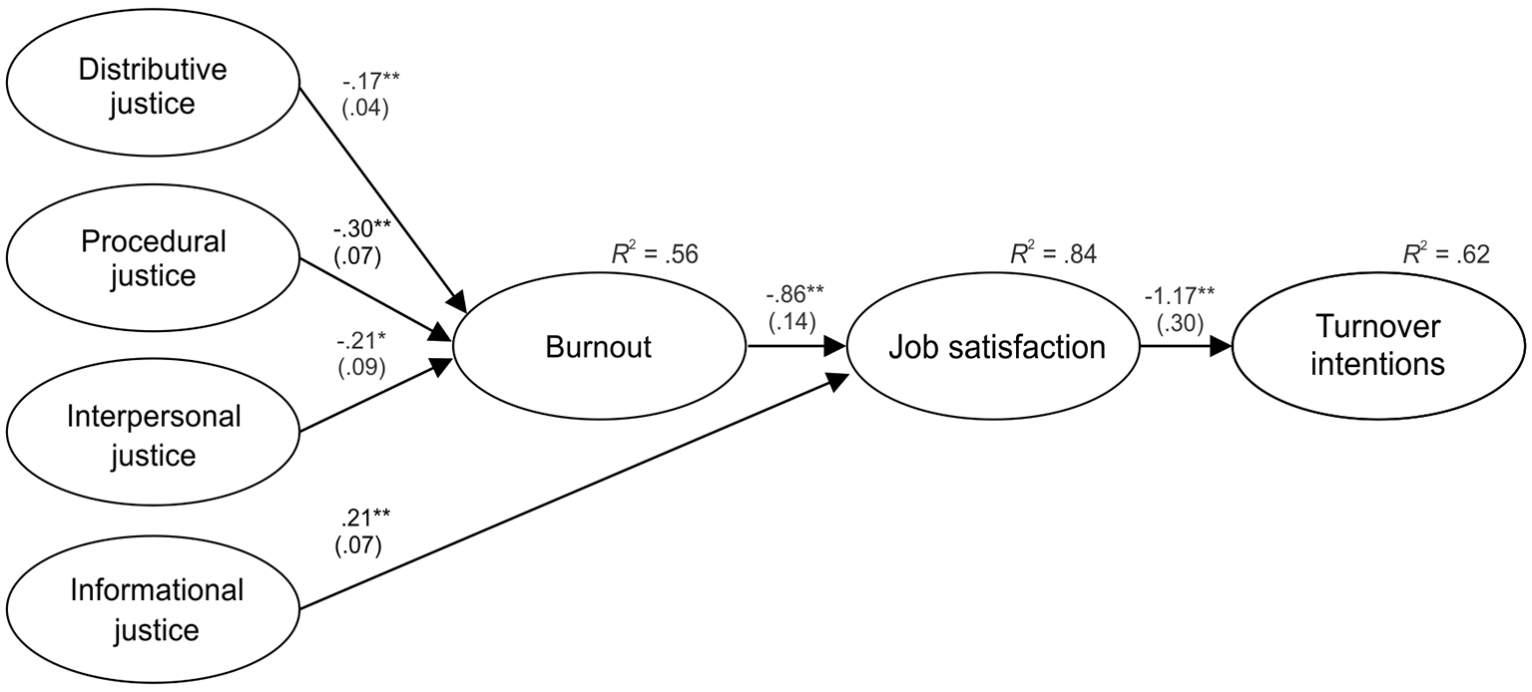
Final serial mediation model. *Note.* Unstandardized regression coefficients (B), standard errors (in brackets), and *R*^2^ are reported. For ease of visual interpretation, observed variables (indicators) and non-significant relationships were eliminated from the figure. *p < .05. **p < .01.

The strategy of rival models was adopted, contrasting two mediation models: a first model in which the direct effects of organizational justice on job satisfaction and TI were restricted to zero, as well as the direct effects of burnout on TI; and a second model that was freely estimated, including all residual direct effects. Although both models showed satisfactory fit indices (Table 4), the results of the χ^2^ difference test (Δχ^2^(9) = 17.88, *p* = .04) indicated that the residual effects added to the second mediation model significantly improved goodness-of-fit. Figure 2 presents the regression coefficients of this second model. As can be seen, distributive, procedural, and interpersonal justice are negatively related to burnout, which is negatively related to job satisfaction, leading to decreased TI among employees. Contrary to expectations, informational justice only presents a positive relationship with job satisfaction, which could be identified by the addition of the residual effects in the second model. The amount of variance explained (*R*^2^) for each endogenous construct was 56% for burnout, 84% for job satisfaction, and 62% for TI.

**Table 4:** Goodness-of-Fit Statistics for the Estimated Mediation Models

Model	*χ*^2^	*df*	*χ*^2^/*df*	CFI	TLI	PNFI	RMSEA	PCLOSE
First mediation model	1188.60**	683	1.74	.95	.95	.83	.04	.99
Second mediation model	1170.71**	674	1.74	.95	.95	.82	.04	.99

*Note.* CFI = comparative fit index; TLI = Tucker-Lewis index; PNFI = parsimony normed fit index; RMSEA = root mean standard error of approximation (RMSEA); PCLOSE = *p* of close fit.

**p < .001.

Finally, bootstrap analysis was performed to check for serial multiple mediation. Table 5 presents the indirect effects calculated with their corresponding 95% confidence intervals. As can be observed, the distributive, procedural, and interpersonal dimensions of organizational justice exert a significant negative indirect effect on TI through burnout and job satisfaction. This implies that perceptions of justice promote lower levels of burnout among employees, which in turn increases their job satisfaction, promoting lower TI. Informational justice only presents a negative indirect effect on TI through job satisfaction. There were no significant residual direct effects of organizational justice on TI. These results supported Hypothesis 4, except for the mediating role of burnout in the relationship between informational justice, job satisfaction, and TI.

**Table 5:** Indirect Effects of Organizational Justice on TI

Relations	Indirect effects	95% bootstrap CI
Distributive justice→Burnout→TI	.01	-.16; .38
Distributive justice→Satisfaction→TI	-.08	-.30; .06
Distributive justice→Burnout→Satisfaction→TI	-.17*	-.64; -.06
Procedural justice→Burnout→TI	.03	-.28; .68
Procedural justice→Satisfaction→TI	.05	-.17; .54
Procedural justice→Burnout→Satisfaction→TI	-.30*	-1.22; -.10
Interpersonal justice→Burnout→TI	.02	-.18; .67
Interpersonal justice→Satisfaction→TI	.11	-.17; .71
Interpersonal justice→Burnout→Satisfaction→TI	-.22*	-.93; -.03
Informational justice→Burnout→TI	.00	-.07; .33
Informational justice→Satisfaction→TI	-.25*	-.88; -.01
Informational justice→Burnout→Satisfaction→TI	-.04	-.35; .21

*Note.* Unstandardized indirect effects are reported (B). CI = confidence intervals; Satisfaction = job satisfaction; TI = turnover intentions.

*p < .05.

## Discussion

The aim of the present study was to test a serial multiple mediation model that explored the mediating role of burnout and job satisfaction in the relationships between organizational justice perceptions and TI of Argentine employees. The development and verification of this theoretical model sheds light on the understanding of a problem that is common to many organizations and involves complex, costly, and time-consuming solutions: workers’ withdrawal cognitions and behaviors.

Our findings show that perceptions of justice are negatively related to TI of employees. According to previous studies (Colquitt, 2001; Colquitt et al., 2013; Flint et al., 2013; Kim & Kao, 2014; Suurd Ralph & Holmvall, 2016), workers who present less TI are those who perceive fairness in salary, promotions, and other outcomes (distributive justice), in the methods and procedures used to arrive at decisions (procedural justice), in the quality of the interpersonal treatment received (interpersonal justice), and in the adequacy and amount of explanations provided by management (informational justice). As can be derived from the postulates of social exchange theory (Blau, 1964), these results suggest that individuals who perceive fairness in their organizational exchanges experience less intentions to quit or withdraw from their jobs, while those who perceive low justice and lack of reciprocity in their relations with the organization, manifest higher TI.

The structural equation analysis indicates that the relationships between organizational justice and TI are mediated by burnout and job satisfaction in an interconnected chain. Specifically, perceptions of distributive, procedural, and interpersonal justice are linked to lower levels of burnout, which results in greater job satisfaction, promoting in turn fewer TI among employees. This finding is consistent with a growing number of studies that have observed that organizational justice negatively impacts on the emotional exhaustion, depersonalization, and diminished personal accomplishment that characterize burnout syndrome (Campbell et al., 2013; Flint et al., 2013; Tayfur et al., 2013). In line with the conservation of resources theory (Hobfoll, 1989), justice perceptions are associated with resources valued by employees (e.g., salary, rewards, fair procedures, adequate interpersonal treatment, etc.), and obtaining and maintaining these resources is related to lower levels of burnout. Prior research (Leka & Jain, 2010; Moura et al., 2014; Scanlan & Still, 2013) has reported that the reduction of burnout syndrome leads to greater job satisfaction, a variable that is recognized not only for its mediating role between work stress factors and TI (Ferreira et al., 2017; Kuo et al., 2014), but also for its potential to decrease withdrawal cognitions in different occupational groups (Allisey et al., 2014). On the contrary, the results suggest that low justice perceptions produce a feeling of lack of control over work conditions and outcomes, thus generating an increase in burnout and a decrease in job satisfaction, which in turn fuels TI among individuals.

Furthermore, perceptions of informational justice show positive associations with job satisfaction, which promotes a reduction of TI. This finding indicates that the provision of adequate, timely, and convenient explanations of the decision-making process acts as a motivator for employees (Colquitt et al., 2013; Silva & Caetano, 2016), reinforcing job satisfaction and strengthening intentions to stay in the organization.

It is worth noting that of the four dimensions of organizational justice, procedural justice had the strongest indirect effect on TI through the proposed mediators. This observation can be explained by the fact that perceived fairness in organizational procedures fosters stronger work emotional responses, providing a basis for the development of relationships of greater trust, loyalty, and involvement with the organization (Colquitt et al., 2013; Tayfur et al., 2013).

The results of the study provide several practical implications for organizations. In this regard, human resource managers and specialists could design and implement different talent retention strategies in order to reduce the undesirable indirect effects of low organizational justice on TI. These strategies can be conceived in terms of distributive, procedural, interpersonal, and informational factors that can optimize employee retention and thus intervene in the withdrawal process before it is too late.

Distributive retention strategies do not only include higher salaries, but also fringe benefits and incentives aimed at recognizing employees’ motivation, performance, and commitment. In fact, the best distributive way of promoting and motivating employees to stay in the organization would be a combination of appropriate pay across organizational levels, career opportunities, promotions, bonus, and other kinds of rewards such as retirement benefits and travel possibilities (Al Mamun & Hasan, 2017; Bryant & Allen, 2013). This is particularly important for employees who possess skills that are in demand, since they are likely to be tempted by higher salaries or better benefits.

There are also several retention strategies organizations can adopt that do not entail major additional costs. These solutions involve procedural, interpersonal, and informational approaches. With respect to procedural strategies, it would be wise to implement fair and equitable procedures for making distributions and compensations as well as developing programs that include job rotation and reduce role ambiguity and role conflict (Bryant & Allen, 2013; Ferreira et al., 2017). Moreover, employers should provide the necessary conditions for employees to meet their knowledge and work-family needs (e.g., positions that match their skills, fair appraisal policies, monitoring levels of work-family balance), and also create opportunities for greater employee participation in the decision-making process (Al Mamun & Hasan, 2017; Colquitt et al., 2013; Deery & Jago, 2015; Silva & Caetano, 2016). What is more, if the organizational culture of Argentine public and private organizations is considered (Omar & Urteaga, 2010), another effective strategy would be to implement more flexible human resource practices and clearer organizational goals in the public sector, and more employee-focused human resource practices and more democratic participation in the private sector. Such strategies would encourage not only fairness perceptions at work, but also a reduction of stress levels and an increase in job satisfaction, favoring employee retention.

As regards interpersonal strategies, human resource experts assert that poor leadership and supervision leads to higher turnover rates. Hence, it is vital for organizations to coach their managers in effective leadership skills and to promote honest respectful interpersonal working relations. In this sense, “one of the most important retention tools is being a leader instead of a manager” (Al Mamun & Hasan, 2017, p. 68), pushing towards the potential of employees, appreciating their performance, and providing tools for personal and professional development (Ferreira et al. 2017). Managers could also create opportunities to foster interaction and cohesion between newcomers and experienced workers, so as to increase the sense of being embedded in the organization. Last, concerning informative retention strategies, management should create work environments where important information is shared efficiently, and standards and procedures used for making organizational decisions are clearly communicated (Bryant & Allen, 2013).

The proposed serial mediation model suggests that if retention strategies are applied, organizations have a good chance of improving organizational justice perceptions, thus reducing the harmful impact of low fairness on burnout, job satisfaction and, eventually, TI. Efforts to improve talent retention should also include mechanisms to periodically assess the mediating variables involved in the turnover process. This practice requires building up basic trust among employees or working with outside consultants to collect, analyze and interpret the data (Bryant & Allen, 2013).

Last, but not least, it is necessary to point out some strengths and limitations of the study. Among the latter, it should be noted that the convenience sampling method prevents the generalization of the results to the population of Argentine workers. Also, the cross-sectional nature of the study implies that no conclusions can be drawn with respect to causal relationships. To overcome these limitations, future research could examine the problem through experimental or longitudinal designs. Nonetheless, it should be highlighted that the direct and indirect effects found in our mediation model fit in a large research tradition and are consistent with both theoretical postulates and previous empirical results (Campbell et al., 2013; Colquitt et al., 2013; Flint et al., 2013; Kim & Kao, 2014; Kuo et al., 2014; Leka & Jain, 2010; Scanlan & Still, 2013). Another possible weakness concerns the use of self-report measures, which could have caused common method bias. To address this possible limitation, some non-statistical remedies were introduced in the research design (Podsakoff et al., 2012), such as verbal and written assurances of response anonymity and the use of distinct sections, instructions, and response formats throughout the questionnaire, all of which probably contributed to the satisfactory result of the Harman’s single-factor test. As for the strengths of the study, to our knowledge this is the first attempt to explore not only TI in the Argentine organizational context, but also the possible intermediate links that act in the relationships between justice perceptions and TI. Our results extend and support past research that has reported significant partial relationships between the variables under study, providing empirical confirmation of the serial multiple mediation model proposed. In particular, this paper extends previous findings (Salessi & Omar, 2017) by verifying the mediating role of job satisfaction in the relationships between certain dispositional/contextual variables and key outcome variables for the functioning of Argentine organizations. Future research could test our model with samples of employees from other countries, as well as consider the inclusion of other potentially significant variables for the development of withdrawal cognitions and behaviors (e.g., organizational commitment, positive/negative affect, organizational support, etc.). Finally, the findings of our study not only contribute to a better comprehension of the processes that lead employees to leave their organizations, but also provide useful information to design and implement organizational programs to reinforce job stability and improve employee retention.
